# Irisin a Novel Metabolic Biomarker: Present Knowledge and Future Directions

**DOI:** 10.1155/2018/7816806

**Published:** 2018-10-09

**Authors:** Ilse Yessabel Martinez Munoz, Eneida del Socorro Camarillo Romero, Jose de Jesus Garduno Garcia

**Affiliations:** ^1^Centro de Investigacion en Ciencias Medicas, Universidad Autonoma del Estado de México, Cuerpo Academico Salud del Universitario, Mexico; ^2^Facultad de Química de la Universidad Autónoma del Estado de México, Mexico; ^3^Hospital General Regional No. 251, Instituto Mexicano del Seguro Social, Mexico; ^4^Fundación IMSS A.C., Mexico

## Abstract

The rising prevalence of chronic diseases such as type 2 diabetes and cardiovascular diseases owing to fat mass excess has been described. In recent years, muscle function/dysfunction has become relevant in metabolic homeostasis. Irisin was described as an exercise-induced myokine. It is the product of type I membrane protein cleavage encoded by the fibronectin type III domain containing 5 (FNDC5) gene. The main beneficial function attributable to irisin is the change of subcutaneous and visceral adipose tissue into brown adipose tissue, with a consequential increase in thermogenesis. Irisin has also been described as a hormone that may have a key role in glucose homeostasis. The way the association of type 2 diabetes with obesity occurs is not fully understood. In recent years, the possible pathways through which irisin could interact with other organs such as the brain or bone have been described. The present paper intends to review the new findings and possible new directions in irisin research.

## 1. Introduction

Obesity is a health problem present in developed and developing countries [1]. Insulin resistance has been considered the link between obesity and chronic degenerative diseases [2]. The rising prevalence of chronic diseases such as type 2 diabetes and cardiovascular diseases owing to fat mass excess has been described [3]. Over the past decade, most of the research efforts related to metabolic diseases have been focused on adipose tissue and its role in chronic inflammation [4]. Recently, muscle function or dysfunction has become relevant in metabolic homeostasis. As it has been previously proposed for gut and adipose tissue, skeletal muscle may be considered an endocrine organ, capable of secreting hormones called myokines [5], highlighting its muscular role in postprandial glucose uptake and lipid metabolism [6].

## 2. Irisin: A Novel Molecule

The first report on irisin was published in 2012 by Bostrom in Harvard University. Irisin was described as an exercise-induced myokine with a peptide structure of 112 amino acids [7]. Irisin is the product of type I membrane protein cleavage encoded by fibronectin type III domain containing 5 (FNDC5) genes [8]. Specifically, the FNDC5 structure consists of a 29-amino acid signaling peptide, a 94-amino acid domain, and a C-terminal, which is considered the lysis performing site prior to being secreted into circulation as irisin. This molecule has been described in other mammals, in which it may have very similar functions as well as structure; for instance, it has a 100% similarity between mice and humans [9].

Irisin is secreted mainly in skeletal muscle, especially in the perimysium, endomysium, and nuclear parts, albeit adipose tissue, pancreas, sebaceous glands, and cardiac muscle have been identified as secretory tissues. Irisin immunoreactivity has been found in salivary glands, ovaries, testes, rectum, intracranial arteries, tongue, optic nerve, stomach, neuronal cells, and sweat glands [9].

One of the most important functions of irisin is the possible regulation of thermogenesis. Vaughan et al. [10] researched on this process in muscle *in vitro*. Irisin acts to increase the expression of the receptor activated by peroxisome ɣ and its coactivator-1*α* (PGC-1*α*), which in turn stimulates the manifestation of intracellular factors with specific functions in mitochondrial biogenesis [10] such as mitochondrial uncoupling protein mRNA 1 (UCP1) [11]. Zhang et al. conducted experiments to elucidate the molecular mechanisms of irisin, finding that r-irisin treatment raises UCP1 through the increased phosphorylation of p38 mitogen-activated protein kinase (p38 MAPK) and regulatory kinases [12]. In this way, irisin is proposed as a hormone capable of increasing energy expenditure, promoting weight loss, and decreasing insulin resistance produced by the diet [8].

Irisin measurement is carried out by enzyme-linked immunosorbent assays in plasma or serum (ELISA) or by the expression of Fndc5 mRNA [13]. According to Huh et al., in muscle, there is also expression in the pericardium, rectum, and heart and it also may be found in the kidney, liver, lungs, and adipose tissue [14]. The validation of both tests has been debated by Albrecht et al., after analyzing the different polyclonal antibodies available in the market to measure irisin concentration and the expression of FNDC5 mRNA [15]. In contrast, Jedrychowski et al. developed a method for the quantification of irisin using the technique of mass spectrometry in tandem, verifying the existence of irisin, making it possible to quantify it with greater accuracy and, in addition, to demonstrate that irisin is present in similar or even higher concentrations than hormones such as insulin, resistin, and leptin [16].

## 3. Irisin and Exercise

Protective myokines are supposed to be secreted over muscle contraction, and this may be the possible link between exercise and protection against chronic diseases and the possible relation of these diseases with physical inactivity [17]. It is well known that physically active lifestyles are protective against T2DM, cardiovascular diseases, cancer, dementia, and depression [18]. Since irisin is a myokine that participates in beneficial processes attributed to exercise and muscle contraction, [8] investigations that link it with different types of physical exercise have been carried out, with no conclusive results nevertheless [19].

One of the first studies on humans was published by Steward et al. (2012); they correlated the expressions of FNDC5 and PGC-1*α* genes with aerobic performance measured through maximal oxygen uptake (VO_2_max) and gas exchange (VE/Vco2) in 24 adult men with heart failure and intolerance to exercise attributed to the symptoms and musculoskeletal disorders characteristic of the disease. A positive and statistically significant correlation between PGC-1*α* and FNDC5 genes and aerobic capacity has been reported, which is consistent with those in the article published by Bostrom et al. [20].

Kim et al. report a positive correlation between irisin and the improvement of manual pressure force and the isokinetic strength of legs in aged women after a 12-week resistance exercise program. The authors above propose irisin as a hormone that prevents the decrement of muscular function associated with advanced ages [21].

Other studies show inverse associations [22, 23]. Kerstholt et al. measured the physical condition by means of a cardiopulmonary exercise test with a cycloergometer. The study included a sample of 740 German adult men and women, finding inverse associations in men between irisin concentration and the peak oxygen uptake, defined as the mean of the highest VO_2_ over 10 seconds of the last minute of the exercise, as well as the maximal power output, in watts, maintained over the last 20 seconds; by contrast, in women, the association was positive, attributing these results to sexual differences [22]. Moreover, Scalzo et al. conducted an intervention study to measure changes in the calculation of irisin and in the expression of the FNDC5 gene after nine high-intensity interval training sessions over a three-week period. Opposite associations were found in women and men, adjudicating these discrepancies to the transcription and translation of FNDC5, production and secretion of irisin, corporal composition, tolerance to exercise, and the role of sex hormones [23].

On the other hand, Norheim et al. show the absence of long-term effects on adipose tissue change, assessed through the expression of UCP1; despite that, they still found positive correlations between FNDC5 and PGC-1*α* mRNA, accompanied by the decrease in circulating irisin after the completion of chronic resistance and strength exercises for 12 weeks. However, in this same study, an increase in irisin concentration after strenuous exercise with a decrease after 2 hours, with no increase in FNDC5 mRNA, was reported [24]. The increase in irisin in response to strenuous exercise has also been documented by Huh et al. who reported a decrease 30 minutes after the end of the exercise, without finding effects after an 8-week training program, attributing the possible short effect of the irisin to homeostasis restoration of adenosine triphosphate (ATP), and once achieved, it decreases to basal concentrations [14].

## 4. Irisin and Type 2 Diabetes

Due to the increase in the prevalence of metabolic diseases related to obesity, including T2DM, many metabolic biomarkers have been studied as possible regulators of glucose homeostasis [25].

Since Bostrom et al. propose to explore the clinical uses of irisin in the treatment of obesity and diabetes, based on the fact that the expression of irisin improves glucose tolerance and decreases fasting insulin in mice [8], researchers from all over the world began to study the link between irisin and DM.

Most published studies show decreased irisin concentrations in patients with T2DM regardless of the time of diagnosis and whether they are undergoing any treatment [26–28] and even lower concentration in the presence of complications of T2DM [29, 30]. Choi et al. found decreasing irisin concentrations in adults with newly diagnosed T2DM compared to those with normal glucose tolerance, showing statistically significant inverse associations between irisin and T2DM development [27]. Similarly, Liu and colleagues found significantly decreased concentrations of irisin in adults with T2DM regardless of age, gender, and BMI, associating their findings with deterioration in the expression of PGC-1*α* in subjects with T2DM [26].

Other studies have shown contradictory effects, which suggests that irisin in patients with T2DM is regulated by different body factors such as glucose and fatty acids. Kurdiova et al. carried out an *in vivo* and *in vitro* study, finding opposite effects in each of them; in the *in vivo* study, irisin and mRNA of FNDC5 in the skeletal muscle and adipose tissue showed to be diminished, albeit myotubes from the *in vitro* study had a greater expression of FNDC5 [28]. A minority of studies indicate the lack of association between irisin and T2DM [13].

Among the relevant clinical aspects in patients with T2DM are the prevention and development of diabetic nephropathy, because they are the main causes of end-stage renal disease [31]. Liu et al. found significantly decreased levels of irisin in patients with T2DM and renal failure, especially in stage 5 of chronic kidney disease, without finding associations with other biomarkers of nephropathy, assigning their results to muscle wasting, insulin resistance, and alterations in energy metabolism related to kidney disease, in addition to the negative association produced by uremic toxins in the expression of FNDC5 [30].

Irisin has displayed to decrease in people with T2DM and macrovascular complications such as coronary artery disease and peripheral vascular and cardiovascular disease, compared to patients without macrovascular complications, proposing this myosin as a possible marker of macrovascular disease in people with T2DM [29].

Taking into account the different types of DM, Ebert et al. published one of the first articles associating gestational diabetes (GD) with irisin; among their main results, they showed that over pregnancy, there are no differences in irisin concentration in the groups of women with GD and healthy pregnant women; irisin was significantly higher in the group of women with GD. The authors also found a positive association of fasting insulin with irisin in women with GD, attributing their findings to possible irisin compensation to counter insulin resistance and to limit its adverse metabolic and vascular effects, as well as a probable resistance to irisin [32]. Consistent with the previous study, Piya et al. describe significantly lower concentrations of irisin in nonobese women without diagnosis of GD compared with those with GD and BMI higher than 30 kg/m^2^; this finding was only shown after adjusting the data for BMI, serum lipids, and glucose, and the conclusion of the authors was the possible resistance to irisin [33]. In spite of this, there is evidence that shows significantly lower concentrations of irisin in women with GD, which attributes these results to the possible damage in the expression of PGC-1*α* and muscle function in women with GD [34].

## 5. Irisin and Type 1 Diabetes

Research in patients with type 1 diabetes has been described, and the evidence is also unclear. T1DM is a multifactorial disorder that is caused by the destruction of pancreatic *β* cells and that involves numerous genetic and environmental factors [35]. Data regarding irisin serum concentration are still controversial. Faienza et al. reported increased irisin levels in T1DM children and adolescents compared to control patients, and they also researched the correlation of irisin and bone metabolism. These authors found a negative correlation between HbA1c and vitamin D in T1DM patients, while a positive one was found with bone mineral density and bone remolder markers evaluated by the BTT-Z score and osteocalcin, respectively. Their results highlighted that in T1DM children and adolescents on continuous subcutaneous insulin infusion, elevated irisin levels predicted a better metabolic control and the possible association through irisin of a better glycemic control and bone health [36].

Ates et al. examined the relation of irisin levels and autoimmunity in T1DM adults. They found higher irisin concentrations in T1DM patients compared to the control group; contrary to the results of Faienza et al., this research reported a positive correlation between irisin and HbA1c and glutamic acid decarboxylase (anti-GAD). In this research, in anti-GAD- and islet cell antibody- (ICA-) positive patients, irisin levels were found higher than those in negative patients [37].

Recently, betatrophin has been described as a hormone secreted by the liver and adipose tissue with the capacity to improve metabolic control in mice by inducing *β* cell proliferation in response to insulin resistance. Espes et al. characterized the levels of irisin in type 1 diabetes and investigated a potential correlation with betatrophin in individuals with T1DM and healthy controls. They reported increased circulating levels of irisin in T1DM patients compared to healthy controls, and the levels of irisin were highest in women with T1DM. A positive correlation was observed between irisin and total betatrophin, but not full-length betatrophin, and the authors suggest that the reason for this may be the differences in betatrophin proteolytic regulation between individuals. In women with T1DM, a negative correlation was observed between irisin and insulin requirements; however, there was no correlation with glucose or HbA1c [38].

## 6. Irisin and Body Mass Index

Irisin has also been related to different anthropometric parameters and body composition, finding discrepancies in different studies [18].

In a study carried out in Spain, Pardo et al. found a higher concentration of circulating irisin in obese people compared to individuals with normal weight and anorexia, reflecting a statistically significant positive correlation between the percentage of fat mass and irisin as well as a negative correlation with fat free mass [17]. In this study, the different types of adipose tissue are proposed as important factors in the secretion of irisin, especially in conditions of obesity; also, this study supports the theory of a possible resistance to irisin [17]. Consistent with the previously mentioned study, Yan et al. found a negative correlation, though not statistically significant (p=0.051), between the amount of muscle mass and irisin concentration in Chinese people with obesity [39].

As regards waist circumference, as an indicator of visceral adiposity, in the same study by Yan et al., it is shown that the concentration of irisin decreases as waist circumference, hip circumference, and A/G ratio increase [39].

## 7. Irisin and Metabolic Syndrome

A metabolic syndrome is a set of conditions that include abdominal obesity, dyslipidemia, high blood pressure, insulin resistance, and increased risk of thrombosis. The underlying condition is insulin resistance [40], which produces alterations in adipose tissue and skeletal muscle that decrease the uptake of glucose, resulting in hyperglycemia [41]. Irisin is a hormone that has the ability to activate beneficial changes in adipose tissue that improve muscle activity; therefore, moderate increases in irisin produce an improvement in insulin resistance induced by a diet [8]. However, studies show that irisin is associated with metabolic biomarkers only in nondiabetic patients [26].

Investigations show negative correlations between glucose and irisin metabolism [39, 42]. In a study on obese Chinese adults, it was found that the decrease in irisin is associated with an increased risk of presenting metabolic syndrome and hyperglycemia, considering it to be protective against insulin resistance because it shows negative associations with fasting insulin and glycosylated hemoglobin [39]. This has also been demonstrated in other populations and age groups; it is the case of the study by Al-Daghri et al. in school-age Saudi boys and girls for which negative correlations were observed with fasting glucose and HOMA-IR [42].

Conversely, there are positive associations between irisin and insulin concentration, fasting glucose, and HOMA-IR [14, 17, 43, 44]. Pardo et al. determined a correlation in women with anorexia nervosa, normal weight, and obesity [17], while Fukushima et al. based their findings on their study of obese men and adult women [43]. Other investigations in addition to finding positive associations with the components of the metabolic syndrome also evince a decrease in adiponectin [14, 44]. Park et al. conducted a study with people with and without metabolic syndrome, demonstrating that the group of people with MetS had higher concentrations of irisin and lower adiponectin, associating increased irisin with a greater amount of fat and lean mass during obesity, as well as the possible compensatory role of irisin or resistance with it [44]. Moreover, Huh et al. consider that irisinemia is due to the deterioration of insulin sensitivity and lipid and glycolytic metabolism, considering a possible feedback mechanism between irisin and adiponectin to increase energy consumption in the adipocytes [14].

On the other hand, there is evidence that points to the absence of significant differences in irisin concentration when comparing it in adult groups with normal weight, overweight, and obesity, with adequate health status, as well as the presence of dyslipidemia and T2DM [13].

In a study by Zhang et al., it was found that the peripheral administration of irisin in mice reduces blood pressure and is proposed as the link between brain, skeletal muscle, adipose tissue, and cardiovascular system connected to one another to modulate energy expenditure and cardiovascular functions [45].

## 8. Irisin and Gender

Depending on sex, lower concentrations of circulating irisin are present in men with obesity and without chronic degenerative diseases than in women [39, 42, 43], and this puts forward a possible irisin secretory mechanism related to women's own body fat distribution [43] and to possible implications of anabolic hormones such as estradiol, which favors the increase in muscle mass and has been positively associated with irisin in middle-aged women regardless of BMI [14].

## 9. Irisin and Cardiovascular System

The presence of the metabolic syndrome doubles the risk of cardiovascular diseases (CVD) such as coronary heart disease and stroke. Taking the reversibility of the components of the metabolic syndrome into account, CVD has a preventable potential mainly via weight control [46].

There are studies that link CVD with irisin [47]; Aronis et al. researched irisin as a predictor of acute coronary syndrome in healthy people, without finding conclusive results; nevertheless, in this same study, it was shown that irisin is a hormone that predicts adverse coronary events in patients with coronary artery diseases under treatment with percutaneous interventions. In this way, decreased concentrations of irisin in this population have a 12-month free survival rate following percutaneous coronary intervention [47].

Irisin has been proposed as prevention and therapy for vascular diseases [48, 49]. Different studies suggest that the phosphorylation of the ERK signaling pathway is one of the molecular mechanisms of irisin action [12, 48, 49]. The mechanisms by which endothelial function is linked to irisin have been studied *in vitro* by Song et al. who administered different concentrations of irisin in human umbilical cord endothelial cells (HUVEC), noting that the administration of 20 nM significantly increases the proliferation of endothelial cells via the extracellular signal-regulated kinase (ERK) pathway. In this same study, it was observed that at the same dose of irisin, the apoptosis induced by high glucose concentrations decreases [49]. Further studies demonstrate proangiogenic effects of irisin at doses of 10 nM to 20 nM, specifically in the process of cell migration and stimulation of capillary structures in HUVEC damaged in *in vitro* studies, associating the increase in the expression of metalloproteinases (MMPs), specifically MMP-2 and MMP-9, in addition to protecting endothelial cells *in vivo* with the activation of the ERK signaling pathway [48].

## 10. Irisin and Cancer

Physical exercise is a protective factor against cancer, and in people with oncological diagnoses, it reduces adverse toxicities and the probability of relapse or death after starting antineoplastic treatments and improves their quality of life, albeit the mechanisms of these are not clear beneficial effects yet [50]. However, before irisin was discovered, Hojman et al. reported that myosin secreted during exercise could inhibit the growth of cells with breast cancer [51]. Therefore, different studies have been carried out with the aim of finding the link between irisin and the development of malignant tumors without finding conclusive results [52, 53]. Moon and Mantzoros reported the absence of effects on cell proliferation and malignant potential of thyroid, esophageal, endometrial, and colon cancer cell lines after being treated *in vitro* with different doses of irisin [53]. Conversely, Gannon et al. revealed the ability of irisin to decrease the number of malignant mammary cells through the induction of apoptosis, in addition to decreasing the viability and migration of these cells, and irisin sensitizes malignant mammary cells for chemotherapeutic treatments such as doxorubicin while decreasing drug uptake, without altering nonmalignant cells; therefore, it could be useful in the adjuvant treatment of some neoplasias [54]. Specifically, in breast cancer, significantly lower levels of irisin have been found in women suffering from the disease compared with healthy women, reporting that the increase in one unit of irisin decreases the probability of breast cancer by 90%, and it is proposed as a possible biomarker with great potential for the detection of this disease [52].

## 11. Irisin and Bone Metabolism

The practice of physical exercise is a measure to maintain a balance in bone formation and resorption and prevent diseases such as osteoporosis and problems of bone metabolism [55]. Not only has a nonmechanical interaction between the bone system and muscles been described, but a biochemical coupling has been described as well, in which muscle is able to secrete molecules that affect bone formation; in this way, some myosins, cytokines, and other bone growth factors involved in the communication between skeletal muscle and bone tissue have been found [56]. In this line, irisin has been proposed as a hormone with a probable therapeutic effect for bone mass gain in osteopenia attributed to diseases or muscular diseases [57].

Anastasilakis et al. studied the association between irisin and osteoporotic fractures in postmenopausal women under treatment with teriparatide, which is a drug that stimulates the activity of osteoblasts and inhibits the apoptosis of osteoblasts, and with denosumab, a drug that works by suppressing osteoclastogenesis. In this study, decreased concentrations of irisin were found in women with osteoporotic fractures, regardless of the type of treatment. The authors of this article discuss the possible impact of muscle mass on their results, which was not measured in the study [58]. Subsequently, Palermo et al. found an inverse correlation between irisin and osteoporotic vertebral fractures in postmenopausal women, regardless of fat and muscle mass and even bone mineral density and physical activity, attributing their results to probable positive effects of irisin on bone quality rather than on bone mass [59].

*In vitro* studies demonstrate that irisin promotes osteoblast differentiation. Colaianni et al. performed a study on myoblasts and myotubes obtained from muscles of previously exercised mice in which the expression of alkaline phosphatase and collagen I was increased, in addition to finding osteoblastogenic effects attributed to an irisin-dependent mechanism [60]. Subsequently, Colaianni et al. conducted an *in vivo* study in which they administered low doses of recombinant irisin to young male mice, observing anabolic actions in the bone mass and mineral density of the cortical tissue and reporting a decrease in osteoclasts and an increase in the expression of osteoblastic genes and a decrease in the expression of osteoblastic inhibitory genes such as SOST, and this same study reports an improvement in bone geometry through an increase in the periosteal perimeter [60]. The signaling pathway by means of which the irisin exerts its osteoblastic effects was studied by Qiao et al. who demonstrated the activation of p38 mitogen-activated protein kinase (p38 MAPK) and extracellular signal-regulated kinase (ERK) [61]. Colaianni et al. performed a research in an animal model and demonstrated that the administration of irisin prevents and restores bone loss and muscle hindlimb atrophy in mice [62].

## 12. Irisin and Brain

Physical exercise has been associated with the reduction of physical and cognitive complications related to central nervous system disorders [63]. Studies demonstrate that the practice of moderate exercise is linked to increased neurogenesis, survival, and neuronal differentiation and migration [64].

There exists evidence that irisin could have some functions in the central nervous system [33, 65]. Dun et al. reported that irisin and FNDC5 are expressed by different cell types, including Purkinje cells in the rodent cerebellum [65]. Subsequently, Piya et al. found irisin in cerebrospinal fluid of humans, and its expression was detected in the neurons of the paraventricular nucleus, where the neuropeptide Y, which is related to appetite regulation, is also expressed, suggesting that it has central metabolic functions in addition to the peripheral metabolic functions already known [33].

Over the last four years, the possible mechanisms of action and effects of irisin in the nervous system have been investigated; an instance is the study on rodents by Li et al. who report that irisin is possibly responsible for the neuroprotection of physical exercise for diseases such as cerebral ischemia, through the activation of ERK1/2 and Akt pathways in brain tissue, as well as protection against brain damage once administered [66]. Moon et al. found that irisin at pharmacological doses increases neurogenesis via the STAT3 signaling pathway, without finding association with the AMPK and ERK pathways *in vitro* [67].

Furthermore, the PGC-1*α*-FNDC5-BDNF-signaling pathway has been proposed for resistance exercise that increases the expression of FNDC5 and in turn induces brain-derived neurotrophic factor (BNDF) [68], which has functions in the transcription and transport of mRNA along dendrites, growth, differentiation, and survival of neurons [69].

Taking into account that fact that irisin promotes favorable processes in the nervous system [66–68] and that there are neurodegenerative disorders such as schizophrenia or major depression related to decreased neurogenesis [64], it is necessary to carry on with research aimed at using the therapeutic potential of irisin in neuronal disorders.

## 13. Conclusions and Future Directions

Muscle has been considered a target organ for many years. Irisin is a novel molecule produced by the muscle. It has been demonstrated that it is related to different metabolic markers. At present, it is not clear which impact of irisin as a possible target in diseases such as diabetes and metabolic syndromes may be. In the future, it will be a challenge to identify a possible clinical application.
